# Urinary extracellular vesicle microRNA profiles and prostate cancer biopsy outcome: Moderate discrimination and quantified optimism under leakage-controlled machine learning

**DOI:** 10.1016/j.bbrep.2026.102820

**Published:** 2026-10-01

**Authors:** Ayoub Slalmi, Nabila Rabbah, Ikram Debbarh, Ilham Battas, Hicham Medromi, Abdelmjid Abourriche

**Affiliations:** a Laboratory of Biology and Health (LBS), Faculty of Sciences Ben M'Sik, Hassan II University of Casablanca, Casablanca, Morocco; b Research Foundation for Development and Innovation in Science and Engineering (FRDISI), Casablanca, Morocco; c Laboratory of Complex Cyber Physical Systems (LCCPS), ENSAM, Hassan II University of Casablanca, Casablanca, Morocco; d Graduate School of Biomedical Engineering and Health Techniques (SUPTECH-SANTE), Mohammedia, Morocco

**Keywords:** Prostate cancer, Urine, Extracellular vesicles, microRNA, Machine learning, Nested cross-validation, Information leakage

## Abstract

**Background:**

Urinary extracellular-vesicle-associated microRNAs are candidates for non-invasive prostate cancer assessment. We evaluated discrimination between GSE159177 records annotated with a Gleason Score and those labelled Control; individual negative-biopsy status could not be confirmed.

**Results:**

The frozen analysis set comprised 465 urinary sample records (278 Gleason-labelled; 187 Control-labelled). Biological annotation retained 2578 mature human microRNA probes from 6658 array probes. Five-fold nested cross-validation confined feature selection, scaling, model-family selection and threshold estimation to outer-training data, yielding one held-out prediction per record. Across five repetitions, mean pooled area under the receiver-operating-characteristic curve (AUC) was 0.707 (sample standard deviation 0.017; range 0.681-0.726). The original seed-42 AUC was 0.726 (95% confidence interval 0.680-0.772), the highest repetition estimate. Its thresholded predictions gave 55.8% sensitivity and missed 123 of 278 positive-labelled records (44.2%). Global outcome-informed feature selection increased AUC by 0.041 (paired 95% confidence interval 0.003-0.080) in the matched experiment, quantifying one source of optimism. hsa-let-7b-5p was the only Mann-Whitney result significant after Benjamini-Hochberg correction and was recurrently selected, but remains exploratory.

**Conclusions:**

This analysis combines biological annotation, leakage-controlled nested evaluation and an empirical leakage comparison to inform independent urinary replication. Discrimination was moderate and partition-dependent. Participant independence remained unresolved without a grouping key; repetitions reused the same records and did not constitute independent validation. Limited sensitivity and imperfect calibration preclude clinical use. Targeted experimental confirmation and participant-level validation are required before clinical translation. Incremental clinical value and economic benefits were not evaluated.

## Highlights

• Leakage-controlled nested reanalysis of 465 urinary EV miRNA records (GSE159177).
• Mean AUC across five recorded splits: 0.707 (SD 0.017; range 0.681-0.726).
• Global label-informed selection inflated AUC by 0.041 (95% CI 0.003-0.080).
• Sensitivity 55.8% with 123 false negatives; calibration slope 0.694
• Hsa-let-7b-5p was the only BH-significant probe; an exploratory candidate only.

## 1 Background

Prostate cancer is one of the most frequently diagnosed malignancies in men and remains a major source of cancer morbidity and mortality worldwide [1]. Serum prostate-specific antigen (PSA) testing can identify men who may benefit from further assessment, but PSA is not cancer specific and does not by itself distinguish benign conditions, indolent tumours and disease requiring intervention. Multiparametric magnetic resonance imaging and targeted biopsy have improved diagnostic pathways, yet imaging performance depends on expertise and equipment, while biopsy remains invasive and subject to sampling limitations. Adjunct tests such as the Prostate Health Index, PCA3 and urine-derived gene-expression scores can refine selected decisions, but their clinical meaning depends on the intended population and endpoint [[2], [3], [4]]. Detection of any cancer, detection of clinically significant cancer, tumour aggressiveness and prognosis are different questions; evidence for one cannot be transferred automatically to another.

Urine is attractive for molecular testing because collection is non-invasive and the urinary tract can contain prostate-derived cells, soluble molecules and extracellular particles. Extracellular vesicles (EVs) carry proteins and nucleic acids and may protect RNA species from degradation [5]. Urinary EV analysis is nevertheless sensitive to collection timing, digital rectal examination, storage, centrifugation, filtration, isolation strategy and normalisation [6]. Current MISEV guidance also cautions against assigning a specific vesicle biogenesis pathway without sufficient physical and biochemical characterisation [7]. The source study associated with GSE159177 described urinary exosome preparations [8,9]. Because vesicle identity and purity can be established only by physical and biochemical characterisation of the preparation itself, we adopt the MISEV-compliant term “urinary extracellular-vesicle-associated” throughout. This wording states exactly what the measurement supports-mature-microRNA abundance in a urinary extracellular-vesicle-enriched fraction-and is what keeps every downstream claim anchored to the specimen actually analysed.

Urinary RNA biomarkers already span different molecular compartments and clinical uses. PCA3 and TMPRSS2-ERG transcripts have been examined in urine or urinary EV fractions, while microRNAs (miRNAs) have been assessed by arrays, sequencing and targeted amplification [[10], [11], [12], [13], [14]]. Some studies have combined urinary RNA signals with PSA or other clinical variables; others have addressed high-grade disease, metastasis or post-operative change rather than biopsy-positive versus biopsy-negative classification [4,15,16]. These studies support biological and technical plausibility, but their AUCs are not directly comparable when analytes, processing, endpoints and populations differ. Serum, plasma and tissue evidence can provide biological context but does not constitute urinary validation. At the level of assay development, a recent systematic and bibliometric review from our group situated multiplex urinary panels within the broader need for analytical validation and clinically defined intended use [17]; that review provides methodological context rather than validation evidence for the present classifier.

Bioinformatic re-annotation is a necessary step before predictive modelling of legacy array data. Versioned probe identities must distinguish mature-human-miRNA probes from precursor miRNAs, other small RNAs and technical controls; otherwise, the biological predictor universe remains ambiguous. The present restriction was therefore anchored to the public GPL21572 record, the source study and the corresponding miRBase annotation rather than inferred from probe numbers alone [8,9,18].

Fig. 1 places the urinary RNA pathway beside a distinct plasma circulating-tumour-DNA pathway. The schematic is contextual, not an output of GSE159177. The miR-21/PTEN/PI3K-AKT association derives from experimental prostate-cancer cell-line work [19]; PCA3 and TMPRSS2-ERG represent established urinary transcript contexts [3,10]; and plasma BRCA1/2 testing belongs to advanced-disease treatment-selection pathways rather than to the urinary diagnostic analysis performed here [20,21]. None of these contextual analytes was claimed to be measured, selected or clinically validated by this reanalysis.

**Fig. 1:**
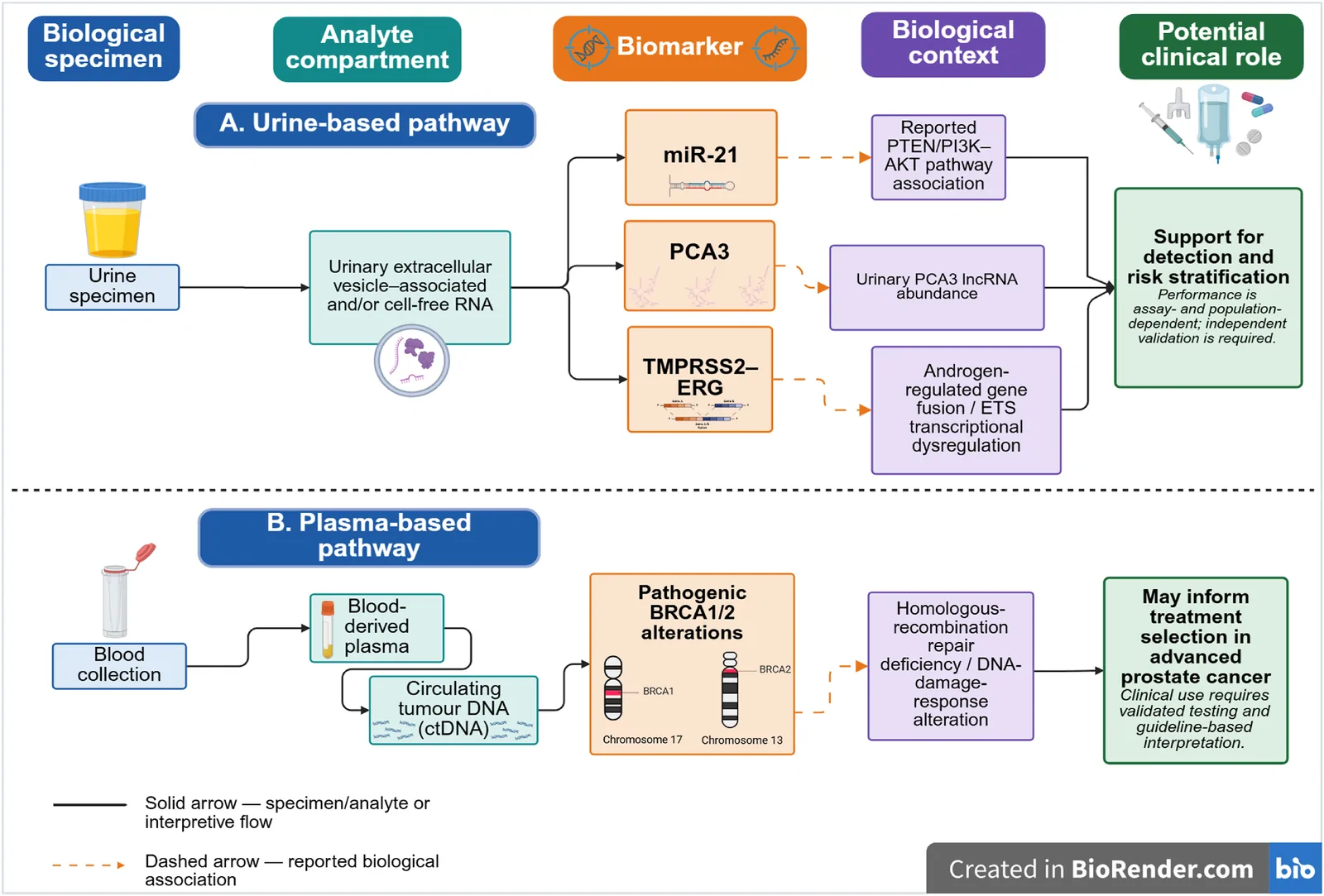
Contextual non-invasive molecular pathways in prostate cancer. (A) Urine-associated extracellular-vesicle and cell-free RNA contexts involving miR-21, PCA3 and TMPRSS2-ERG, with reported relevance to detection or risk stratification. (B) Plasma circulating tumour DNA containing pathogenic BRCA1/2 alterations, shown separately as an advanced-disease treatment-selection context. Solid arrows denote specimen, analyte or interpretive flow; dashed orange arrows denote reported biological associations. The schematic maps the literature-reported relationships that motivate the urinary analytical strategy evaluated here and situates panel A, the compartment interrogated in this study, against the distinct plasma pathway of panel B.

High-dimensional transcriptomic prediction introduces a separate methodological risk. With thousands of candidate probes and only hundreds of observations, variable selection can adapt strongly to a particular dataset. Selecting features globally before cross-validation lets outcome information from nominally held-out observations influence the model and inflates apparent performance [22,23]. Reusing the same validation results for model selection and performance estimation can cause similar optimism [24]. A leakage-controlled evaluation must therefore repeat every data-dependent development step inside training data and reserve each outer-held-out set for a single evaluation of the complete procedure.

We developed and internally evaluated a leakage-controlled machine-learning procedure for prostate-cancer detection from urinary EV-associated mature-miRNA profiles, using a biologically restricted feature universe and nested cross-validation. The primary objective was to estimate discrimination from one outer-held-out prediction per record. Secondary objectives were to assess probability accuracy, calibration, threshold-dependent performance, empirical leakage inflation, feature-selection stability, algorithm-family behaviour, partition sensitivity and a strictly conditional cross-fluid transportability experiment. Descriptive differential analysis was kept separate from predictive feature selection. Throughout, association, predictive recurrence, analytical validation, functional validation and clinical usefulness are treated as distinct levels of evidence [25].

## 2 Methods

### 2.1 Study design, reporting framework and endpoint

This was a retrospective secondary analysis of public expression data. Reporting was guided by TRIPOD + AI for prediction-model studies, STARD only where compatible with diagnostic-accuracy reporting, PROBAST + AI for risk-of-bias considerations and MISEV2023 for EV terminology [7,[26], [27], [28]]. The analysis establishes the internally validated performance of the model-development procedure and provides a traceable basis for subsequent prospective clinical evaluation, independent urinary validation and development of a locked assay.

Every predictor analysed here is a mature-microRNA measurement from total RNA isolated from a urinary exosome preparation. The operational outcome was assigned from the public GEO metadata: records annotated with a Gleason Score were mapped to the positive class and records annotated Control to the comparator class. Supplementary Table S1 links each GSM accession to its original sample title, phenotype metadata and analysis label. Individual negative-biopsy status could not be independently verified from the available metadata.

The endpoint is the binary source-derived label described above. We do not describe the comparator group as healthy controls or assume that every control entered a biopsy-referral pathway. The class assignments and archived predictions were retained without relabelling. Positive/negative and biopsy-positive/biopsy-negative labels in archived analysis files and figures denote these operational classes, not individually reverified pathology outcomes. The public metadata did not support a separate endpoint for clinically significant cancer, grade group, progression or prognosis; these endpoints are the object of the participant-level cohort described in Sec. 4.8. The classifier used molecular measurements without clinical covariates. Its evaluation therefore concerns the urinary expression profiles within the deposited cohort; incremental value beyond PSA, age or MRI was not evaluated, and tumour-specific biology cannot be separated from technical or population effects.

### 2.2 Data source and cohort reconciliation

Expression data originated from NCBI Gene Expression Omnibus accession GSE159177 and Affymetrix GeneChip miRNA 4.0 platform GPL21572 [8,9]. The biological source declared in that submission is urine: total RNA was isolated from urinary exosome preparations and small non-coding RNAs were then profiled on the array. The frozen matrix contained 465 unique GEO sample (GSM) records, each corresponding to one urine specimen: 278 records annotated with a Gleason Score and 187 records labelled Control, an observed case fraction of 278/465 (59.8%). Direct inspection confirmed 465 distinct GSM accessions and 3,095,970 numeric finite expression values across 6658 probes, that is a complete matrix with no missing entry. No sample was excluded on the basis of its expression profile or outcome, so the analysed set is the full public cohort rather than a subset selected after inspection.

The predictive input was the publicly deposited processed expression matrix, not newly processed raw CEL intensities. Inspection of all 465 public CHP headers recorded Transcriptome Analysis Console 4.0.1.36, rma-bg/pm-only/med-polish signal processing, DABG detection and log2-scale signals. All recorded commands used the analysis name rma-dabg-no-norm; no quant-norm operation was present in those commands. We therefore do not describe the input as a complete quantile-normalised RMA pipeline or as spike-in-normalised data. Background correction and signal summarisation were recorded, whereas additional processing outside the recorded workflow remains unconfirmed. Fold-local predictor scaling is separate from array-level normalisation.

The 465 × 6658 CHP expression values matched the deposited matrix after rounding to its six-significant-digit precision. This is a provenance check, not evidence of measurement reliability. Scan timestamps ranged from 2 October 2017 to 18 March 2020 across 13 distinct calendar months; nine months contained only one outcome class. Two processing-directory groups comprised 232 and 233 records. These timestamps and directories are technical metadata, not verified recruitment sites or extraction/hybridisation batches. The recovered technical fields and their class counts are provided in Supplementary Table S1 and its accompanying documentation. No batch correction or new model evaluation based on these fields was performed.

Fluidics records were available in 313 headers; seven recorded a status other than Completed. Their interpretation and any subsequent rerun history are unknown. These events were documented rather than used as automatic exclusion criteria. Matrix completeness, repeated sample titles and technical-header agreement cannot establish specimen quality or participant independence.

The public submission contains sample-level GSM accessions but no participant-grouping key. Consequently, 465 distinct accessions do not establish 465 independent people. If repeated specimens or technical reruns from a participant crossed training and evaluation partitions, performance could be optimistic and record-level intervals could understate uncertainty. The present validation is therefore conditional on record-level splitting and cannot be described as participant-independent. Participant-level linkage was requested from the original depositors but remained unavailable. The meaning of the R1 suffix, including the MU01-1365/MU01-1365R1 pair, remains unconfirmed; no participant identities were inferred from titles. Table 1 summarises the cohort and Supplementary Table S1 records the available provenance.

**Table 1:** Dataset, cohort and feature annotation.

Item	Value	Interpretation or provenance
Data source	GEO GSE159177	Public urinary small-RNA array dataset linked to Wang et al. [8,9]
Specimen analysed	Urine	Total RNA isolated from urinary exosome preparations; no biopsy tissue was analysed.
Outcome-label provenance	Public source annotations	Gleason Score metadata map to the positive class and Control metadata to the comparator class. Individual negative-biopsy status of controls is unconfirmed; no biopsy tissue predictor was used.
Source terminology	Urinary exosome preparation	Reported here as “extracellular-vesicle-associated” in line with MISEV2023, which requires physical and biochemical characterisation before a biogenesis-specific term is used [7].
Platform	Affymetrix GeneChip miRNA 4.0; GPL21572	Frozen expression matrix and platform annotation.
Analysed records	465 unique GSM records	278 Gleason-labelled and 187 Control-labelled records; observed positive-class fraction 59.8%. Source labels do not independently verify individual pathology results.
Participant identifiers	Not released in the public submission	GSM accessions identify records, not necessarily different people [8]. No donor key is available; participant overlap across partitions and underestimated uncertainty cannot be excluded.
Expression completeness	3,095,970 of 3,095,970 values present (100%)	All 6658 probes × 465 records are finite and numeric; the matrix is complete, so no imputation rule was required.
Raw feature universe	6658 probes	Complete array universe, used only in the legacy technical sensitivity analysis.
Primary feature universe	2578 probes	One-to-one mapping to 2578 unique mature human miRNAs in GPL21572/miRBase v20.
Precursor miRNAs excluded	2025 probes	Removed before primary modelling.
Other small RNAs excluded	2006 probes	Human small-RNA probes not annotated as mature miRNAs.
Controls and spike-ins excluded	49 probes	Technical features removed from the primary biological matrix.
Outer validation structure	Five stratified partitions	372 outer-training and 93 outer-held-out records per iteration; each record held out exactly once.

### 2.3 Biological annotation and feature universes

The complete technical array universe comprised 6658 probes. Probe identities were assigned from the frozen GPL21572 annotation linked to miRBase version 20 [18]. For the primary biological analysis, we retained 2578 probes with a one-to-one mapping to 2578 unique mature human miRNA identifiers. The annotation filter excluded 2025 precursor-miRNA probes, 2006 probes representing other human small-RNA categories and 49 controls or spike-ins. These categories reconcile exactly to the 6658 probes, and outcome labels were not used during annotation.

The broad 6658-probe matrix was retained only for a legacy technical sensitivity analysis. It was not allowed to replace the mature-miRNA universe after performance inspection. A numeric probe identifier was never converted to a named miRNA without the frozen, versioned mapping.

### 2.4 Leakage-controlled nested validation

The primary evaluation used five stratified outer partitions created with shuffling and random seed 42. In each iteration, 372 records formed the outer-training set and 93 formed the outer-held-out set. Every record appeared in an outer-held-out set exactly once. The five assignments were frozen and reused for the fixed-family comparisons.

All data-dependent operations were confined to the corresponding outer-training set (Fig. 2). Within that set, five-fold stratified inner cross-validation defined model-family selection and threshold estimation. Feature scoring, selection of 50 probes and scaling were fitted separately within inner-training subsets. Outer-held-out records did not contribute to feature processing, scaling, family selection, model fitting or threshold estimation. After selection, the complete pipeline was refitted on the full outer-training set, locked, and applied once to the untouched outer-held-out records. Probabilities were pooled only after all five iterations were completed.

**Fig. 2:**
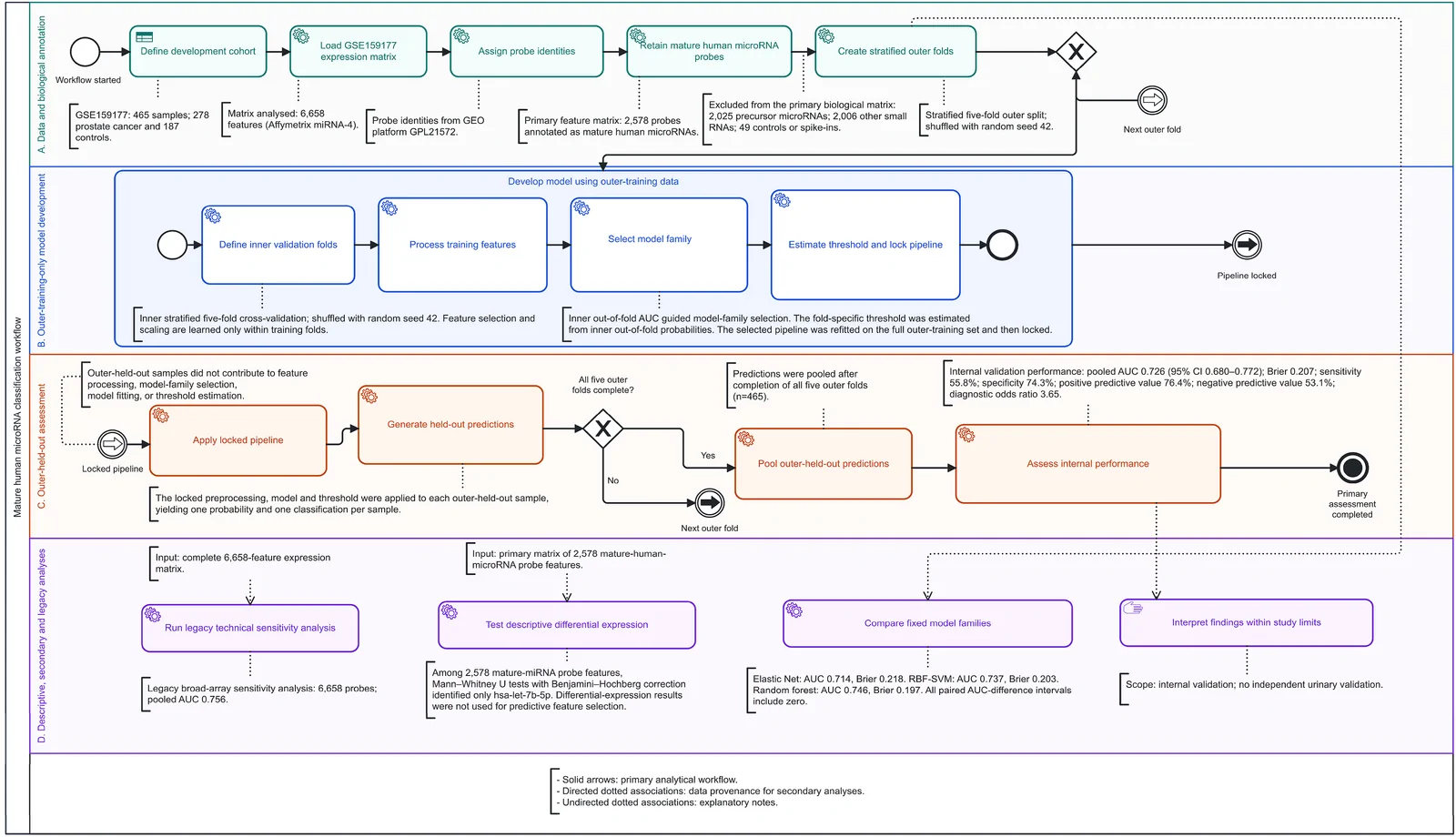
Leakage-controlled mature-miRNA classification workflow. The GSE159177 expression matrix comprised 465 sample records and 6658 array probes. Probe annotation retained 2578 mature human miRNA probes for the primary analysis. Within each outer-training partition, feature processing, model-family selection and fold-specific threshold estimation used inner cross-validation. The fitted pipeline and threshold were then locked and applied to the untouched outer-held-out partition. Predictions were pooled only after completion of all five outer iterations. Legacy broad-array, descriptive differential-expression and fixed-family benchmark branches remained secondary and did not influence the primary held-out predictions.

### 2.5 Primary model-family selection

Each candidate was implemented as a scikit-learn pipeline containing univariate ANOVA F-statistic ranking, selection of the top 50 probes, standardisation and a classifier [29]. The primary procedure compared four prespecified fixed configurations rather than a post hoc hyperparameter search: $L_{2}$-penalised logistic regression (C=1), radial-basis-function support-vector machine (RBF-SVM; C=1, gamma set to scale), random forest (100 trees, square-root feature sampling) and XGBoost with the archived implementation settings [[30], [31], [32]]. Random components used seed 42 and one computational thread where recorded.

For each candidate family, inner out-of-fold probabilities were assembled across the outer-training records and used to calculate an inner AUC. For outer iteration k, the selection rule was

(1) $${\hat{m}}_{k}=\underset{m\in \{\text{LR},\text{RBF}‐\text{SVM},\text{RF},\text{XGB}\}}{\mathrm{argmax}}\;\text{AUC}\left(y_{k}^{\text{train}},{\hat{p}}_{k,m}^{\text{inner}‐\text{OOF}}\right)\text{,}$$

where ${\hat{p}}_{k,m}^{\text{inner}‐\text{OOF}}$ denotes predictions generated without training on the corresponding inner-validation records. An exact tie was resolved by the prespecified candidate order: logistic regression, RBF-SVM, random forest and XGBoost. The selected family was refitted on the complete outer-training set. No outer-held-out result was inspected when choosing a family or configuration.

### 2.6 Fold-specific threshold estimation

The classification threshold for each outer iteration was estimated exclusively from the selected family's inner out-of-fold probabilities. The threshold maximised Youden's index,

(2) J(c)=Sensitivity(c)+Specificity(c)−1,

where c denotes a candidate probability threshold. The chosen value was locked with the refitted pipeline and applied only to the corresponding outer-held-out probabilities. Threshold performance therefore evaluates a development strategy, not a single universal cutoff.

### 2.7 Primary performance measures and uncertainty

The original primary estimand was the pooled ROC AUC from one outer-held-out probability per record under seed 42. We retain that estimate for traceability and foreground the arithmetic mean and sample standard deviation of the five repetition-specific pooled AUCs in the revised presentation. This repeated mean is distinct from the mean of the five fold AUCs within seed 42; no seed-42 confidence interval is assigned to the repeated mean. Probability accuracy was summarised by the Brier score,

(3) $$\text{Brier}=\frac{1}{n}\sum_{i=1}^{n}{\left(p_{i}-y_{i}\right)}^{2}\text{,}$$

where $p_{i}$ is the outer-held-out probability and $y_{i}$ the binary source label. Internal calibration was described using a reliability plot, probability distributions, and logistic recalibration intercept and slope [33].

Threshold-dependent metrics were computed after applying the five fold-specific thresholds. We report confusion counts, sensitivity, specificity, positive predictive value (PPV), negative predictive value (NPV), accuracy, balanced accuracy and diagnostic odds ratio (DOR),

(4) $$\text{DOR}=\frac{TP\times TN}{FP\times FN}\text{.}$$

Wilson intervals were used for sensitivity, specificity, PPV and NPV. The DOR interval was obtained on the log scale without continuity correction.

Scalar AUC and Brier-score intervals used 5000 stratified record-level percentile-bootstrap repetitions with seed 42; positive and negative records were resampled separately [34]. The complete nested procedure and partition generation were not rerun in each bootstrap sample. These intervals therefore quantify record-level uncertainty conditional on the frozen outer partition, not total development uncertainty. The graphical ROC band used 2000 stratified bootstrap repetitions and is not the source of the scalar AUC interval.

### 2.8 Exploratory decision-curve analysis

Decision-curve analysis (DCA) compared the primary predictions with treat-all and treat-none strategies [35]. At threshold probability $p_{t}$, net benefit was calculated as

(5) $$\text{NB}\left(p_{t}\right)=\frac{TP\left(p_{t}\right)}{n}-\frac{FP\left(p_{t}\right)}{n}\frac{p_{t}}{1-p_{t}}\text{.}$$

The principal display used thresholds 0.05-0.50 and the extended display used 0.05-0.90. Both used the observed 59.8% case fraction without prevalence correction. No clinical threshold was selected from DCA, which remained an internal exploratory analysis.

### 2.9 Descriptive differential analysis

For each of the 2578 mature-miRNA probes, a two-sided Mann-Whitney *U* test compared positive-labelled and control-labelled records. Benjamini-Hochberg (BH) adjustment controlled the false-discovery rate across all tests at 0.05 [36]. The displayed effect was the median expression difference on the log2 scale (positive minus negative). A stratified bootstrap provided a 95% interval for rank-biserial correlation. Welch's test and an independent Python empirical-Bayes moderated-t approximation were sensitivity analyses. The latter was not Bioconductor limma and is not described as limma-equivalent. Differential results were not used for predictive feature selection.

### 2.10 Fixed-family benchmark and algorithm behaviour

Elastic Net, RBF-SVM and random forest were compared as a secondary benchmark using the same frozen outer assignments. Elastic Net combines $L_{1}$ and $L_{2}$ penalties [37]; the RBF-SVM and random forest follow their standard formulations [30,31]. In this benchmark, feature processing and inner configuration search were repeated within each outer-training set. Each family produced its own probability for every outer-held-out record. AUC, Brier score, internal calibration and threshold-dependent metrics were calculated separately. Paired bootstrap differences used aligned held-out records; superiority was not claimed when the interval included zero.

Partition-level AUCs, selected configurations and pairwise Jaccard similarity of selected 50-probe sets were retained as descriptive diagnostics. They do not constitute inferential tests. The primary procedure selects among four families, whereas the benchmark holds each of three families fixed; their results answer different questions and use different tuning budgets. The RF benchmark AUC of 0.746 cannot be interpreted as an improvement over the primary AUC of 0.726 attributable to model family alone.

### 2.11 Selection stability and held-out importance

For the seed-42 primary split, we counted how often each probe appeared among the 50 selected probes across five outer-training sets and calculated pairwise Jaccard overlap. We summarised probes appearing in at least three, four and all five sets. Stability of selection was kept distinct from predictive importance and biological validation [38].

Held-out permutation importance was calculated within the appropriate outer-held-out records as the decrease in AUC after permuting one selected probe, preserving the separation between model fitting and interpretation [39]. Results were aggregated descriptively across partitions. Grey cells in the stability display denote that a probe was not selected in the corresponding outer-training set; this differs from a selected probe whose importance was near zero. Affymetrix probe identifiers were retained unless a unique frozen mapping was available.

### 2.12 Empirical leakage experiment

To quantify the effect of a common invalid practice, we repeated a matched analysis in which the top 50 probes were selected globally using all outcome labels before cross-validation. The same frozen outer partitions were then used. This procedure is deliberately incorrect because the outer-held-out outcomes influence the feature set. It was analysed only as a methodological stress test, never as an alternative model. Paired bootstrap intervals compared its AUC and Brier score with a clean rerun of the strict procedure.

The clean rerun produced AUC 0.726176, whereas the archived canonical predictions produced 0.726196. The difference ($1.9\times 10^{-5}$) resulted from archived probability serialisation; maximum per-record probability deviation was below $5\times 10^{-7}$. The archived pooled value remains the primary reported estimate, while the rerun value is used for the paired leakage comparison and the seed-42 entry of the recorded partition-sensitivity summary.

### 2.13 Partition sensitivity

The entire nested procedure was repeated for five recorded outer-split seeds: 42, 101, 202, 303 and 404. Each repetition generated one held-out prediction per record, but predictions for the same record across seeds were not treated as independent observations. We report seed-specific AUC, Brier score, mean partition AUC and selected-family counts as sensitivity to partition construction rather than as repeated validation cohorts.

Public sample-title prefixes were MU01 (448 records), ISPU-11 (14) and MU03 (3); their interpretation as recruitment sites was not independently verified. All 187 control-labelled records had the MU01 prefix. The post hoc MU01-restricted analysis re-evaluated the frozen held-out probabilities without refitting, reselection or rethresholding. It is a descriptive restriction, not a test that can separate provenance from outcome across groups lacking controls.

### 2.14 Legacy broad-array sensitivity

An archived procedure using all 6658 probes was retained as a technical sensitivity analysis. Because its feature universe includes precursor miRNAs, other small RNAs and technical controls, its AUC cannot replace the 2578-probe primary biological result. It was not selected after comparison and is reported only to quantify dependence on feature-universe definition.

### 2.15 Conditional cross-source transportability experiment

A conditional stress test used GSE112264, a serum-miRNA dataset linked to Urabe et al. [40,41]. This is not an independent urinary validation. After versioned annotation, 2539 unambiguous MIMAT identifiers were shared between source and target matrices. Fifty source-selected features were used to fit logistic-regression and random-forest classifiers on GSE159177 and evaluate 1091 labelled target records (809 prostate-cancer and 282 comparator records). Naive transfer was compared with correlation alignment (CORAL). Target labels were reserved for evaluation and did not train the source model or alignment. Four individual probability vectors were archived. Stratified bootstrap intervals were calculated for each target AUC and paired CORAL-minus-naive AUC difference.

### 2.16 Software and reproducibility

The analysis is bioinformatic before it is predictive, and both layers are versioned. Probe-level re-annotation relied on the frozen GPL21572 platform record and the corresponding miRBase release to resolve mature-miRNA identity [18]; statistical testing used SciPy and multiple-testing control followed Benjamini-Hochberg [36]; model fitting, feature ranking and permutation importance used scikit-learn [29] with XGBoost for the gradient-boosted candidate [32]; matrix handling used pandas and NumPy, and figures were produced with Matplotlib. Seeds, frozen outer assignments, individual predictions, selected features, thresholds, metrics, source tables and figure scripts accompany this manuscript. A clean rerun reproduced all 15 fixed-family benchmark refits within the recorded numerical tolerance. Relative paths and SHA-256 manifests support traceability of the packaged inputs and outputs and inspection of the archived computational results.

This study is positioned at the computational stage of biomarker development [25]. The internal performance estimates, explicit leakage comparison and recurrent candidates provide a structured basis for planning independent urinary replication. Section 4.8 outlines the subsequent assay and EV-characterisation steps needed to evaluate these candidates experimentally and assess their clinical relevance.

## 3 Results

### 3.1 Cohort and annotation reconciliation

All 465 GSM records passed the frozen input checks and appeared exactly once in the primary outer-held-out prediction file. The source-derived class counts were 278 Gleason-labelled and 187 Control-labelled records. All probabilities were within [0,1], and no critical identifier, label, partition, probability, threshold or class prediction was missing.

Biological annotation retained 2578 mature-human-miRNA probes from the 6658-probe matrix. The 4080 excluded probes comprised 2025 precursor miRNAs, 2006 other small RNAs and 49 controls or spike-ins. Fig. 2 shows that this annotation preceded splitting while remaining independent of outcome labels.

### 3.2 Primary family selection and discrimination

The primary inner procedure selected random forest for outer-held-out partitions 1 and 2, RBF-SVM for partitions 3 and 5, and logistic regression for partition 4. XGBoost was evaluated in all training sets but was not selected. The five outer-held-out AUCs were 0.704, 0.737, 0.702, 0.768 and 0.736. Their arithmetic mean was 0.730, a fold-level descriptive average distinct from the pooled record-level estimate.

Pooling the 465 unique outer-held-out probabilities produced an AUC of 0.726 (95% CI 0.680-0.772; Table 2; Fig. 3). The Brier score was 0.207 (95% CI 0.190-0.225). Across all five recorded partition seeds, mean pooled AUC was 0.707 (sample SD 0.017; range 0.681-0.726; Sec. 3.9). The seed-42 result was the highest of these five, not a partition-invariant estimate. Its bootstrap interval does not describe uncertainty in the repeated mean.

**Table 2:** Primary outer-held-out performance.

Measure	Estimate	Uncertainty or dispersion	Estimation note
Seed-42 pooled ROC AUC	0.726	0.680-0.772	Original primary estimate; highest of five recorded repetitions. Repeated mean 0.707, sample SD 0.017 (Table 3); this CI applies to seed 42 only.
Mean of five fold AUCs within seed 42	0.730	SD 0.027; range 0.702-0.768	Mean of five fold-level ratios rather than a record-level estimand; dispersion is therefore reported.
Brier score	0.207	0.190-0.225	Pooled outer-held-out probabilities.
Calibration intercept	0.073	Point estimate	Logistic recalibration on pooled internally held-out predictions.
Calibration slope	0.694	Point estimate	Internal calibration summary; not externally validated.
True positive/true negative	155/139	Observed counts	Exhaustive classification of the 465 held-out records at fold-specific thresholds learned in outer-training data; intervals are reported for the derived proportions.
False positive/false negative	48/123	Observed counts	Completes the confusion matrix: 155+139+48+123=465.
Sensitivity	55.8%	49.9-61.5%	Denominator 278.
Specificity	74.3%	67.6-80.1%	Denominator 187.
Positive predictive value	76.4%	70.1-81.7%	Conditional on observed case fraction.
Negative predictive value	53.1%	47.0-59.0%	Conditional on observed case fraction.
Diagnostic odds ratio	3.65	2.43-5.47	Log-scale interval; no continuity correction.

For the seed-42 analysis, AUC and Brier-score intervals used 5000 stratified record-level bootstrap repetitions. They quantify uncertainty conditional on the frozen outer partition because the complete nested procedure was not rerun in each bootstrap sample. Calibration intercept and slope are descriptive internal point estimates from pooled outer-held-out predictions; uncertainty intervals were not prespecified. Predictive values reflect the observed 59.8% case fraction and are not population-prevalence estimates.

**Table 3:** Robustness, stability and transportability analyses.

Analysis	Result	Interpretation
Incorrect global feature selection	AUC 0.768 versus strict rerun AUC 0.726; paired AUC difference +0.041 (95% CI +0.003 to +0.080)	Empirical demonstration of optimistic bias when labels from evaluation records influence the feature set; not a competing model.
Paired Brier difference, leaky minus strict	−0.013 (95% CI −0.030 to 0.003)	Direction favoured the invalid procedure, but the interval included zero.
Primary feature-selection stability	42 probes selected in at least 3/5 outer-training sets; 34 in at least 4/5; 27 in 5/5	Descriptive stability of the seed-42 split, not biological validation.
hsa-let-7b-5p predictive status	Selected in 5/5 seed-42 outer-training sets and 25/25 across five seeds; Elastic Net mean coefficient −0.434; mean held-out AUC decrease 0.019 (RBF-SVM) and 0.018 (RF)	Predictive recurrence is distinct from differential association and does not validate a biomarker.
Partition sensitivity	Five-seed AUC range 0.681-0.726; mean 0.707; SD 0.017	The seed-42 result is conditional on its recorded split. The repeated mean describes internal split sensitivity, not independent replication; no CI for that mean is claimed.
Conditional urine-to-serum transfer	LR AUC 0.541 naive and 0.552 CORAL; RF AUC 0.543 naive and 0.578 CORAL	Cross-fluid, cross-platform stress test; not independent urinary validation.
CORAL-minus-naive paired AUC differences	LR +0.011 (95% CI −0.009 to 0.030); RF +0.035 (−0.0003 to 0.070)	Both intervals included zero; no established transportability gain.

**Fig. 3:**
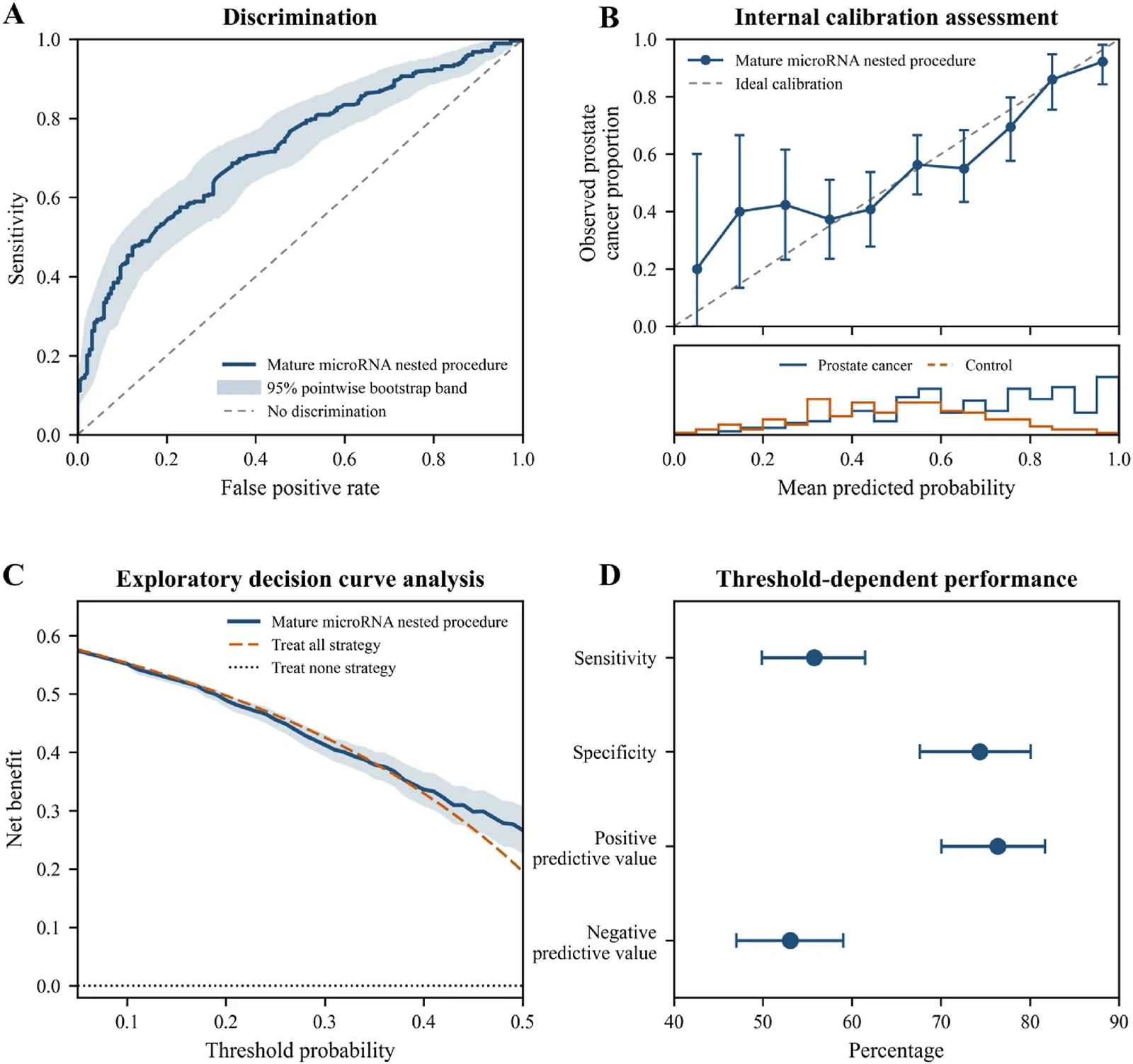
Original seed-42 outer-held-out predictive performance. (A) Pooled ROC curve from one held-out probability per record, with a stratified bootstrap band. (B) Internal calibration by probability bins with distributions for the source-derived positive and control classes. Biopsy-positive/biopsy-negative labels in the archived plot refer to these operational classes; individual negative-biopsy status was not confirmed for controls. (C) Exploratory DCA over the prespecified principal threshold range. (D) Sensitivity, specificity, PPV and NPV with 95% intervals after applying fold-specific thresholds learned within outer-training data. The scalar AUC was 0.726 (95% CI 0.680-0.772), mean partition AUC was 0.730 and Brier score was 0.207. Predictive values and DCA are conditional on the observed 59.8% case fraction.

### 3.3 Calibration and threshold-dependent classification

The exploratory calibration intercept was 0.073 and slope 0.694. Reliability bins showed deviations from the ideal diagonal, particularly where few records contributed. A slope below one is compatible with predictions that are too variable, but this internal summary was not independently validated.

The five inner-derived thresholds were 0.600, 0.640, 0.763204, 0.540265 and 0.670024. Applying each value only to its corresponding outer-held-out probabilities yielded 155 true positives, 139 true negatives, 48 false positives and 123 false negatives. Sensitivity was 55.8% (95% CI 49.9-61.5%), specificity 74.3% (67.6-80.1%), PPV 76.4% (70.1-81.7%), NPV 53.1% (47.0-59.0%) and DOR 3.65 (2.43-5.47). Accuracy was 63.2% and balanced accuracy 65.0%. The 123 false negatives and the case-enriched sample composition preclude a clinical-use claim.

### 3.4 Empirical effect of information leakage

Leakage is usually invoked qualitatively; here it was measured. Repeating the entire analysis with the top 50 probes chosen globally before cross-validation-an invalid outcome-informed selection strategy-raised the AUC from 0.726 to 0.768 on identical outer assignments. The paired increase was 0.041 (95% CI 0.003-0.080; Table 3), and the Brier score moved from 0.207 to 0.194, a paired difference of −0.013 (95% CI −0.030 to 0.003). This paired contrast estimates the effect of changing that selection step within the recorded matched experiment; it does not quantify every source of optimism.

The leakage contrast should be interpreted alongside other analytic sensitivities. The five recorded splits produced an AUC range of 0.681-0.726, with mean 0.707 and sample SD 0.017. The broad-array sensitivity yielded AUC 0.756, but used a different feature universe. These are different estimands and designs: a between-seed range or a difference between feature universes is not a paired causal estimate of leakage. The +0.041 result does not establish that leakage dominates partition variability, tuning choices, technical confounding or participant dependence, and cannot be generalised as the amount of bias in the wider literature.

### 3.5 Exploratory hsa-let-7b-5p signal

Among 2578 Mann-Whitney tests, hsa-let-7b-5p was the only result with BH-adjusted p<0.05 (adjusted p=0.0142). Its positive-class-minus-control median difference was −0.163 on the log2 expression scale. The rank-biserial effect was negative, and the stratified-bootstrap interval did not cross zero (Fig. 4). Under alternative statistics, the same probe ranked 15th by Welch's test and ninth by the independent Python moderated-t approximation, demonstrating statistic-dependent ranking despite concordant direction.

**Fig. 4:**
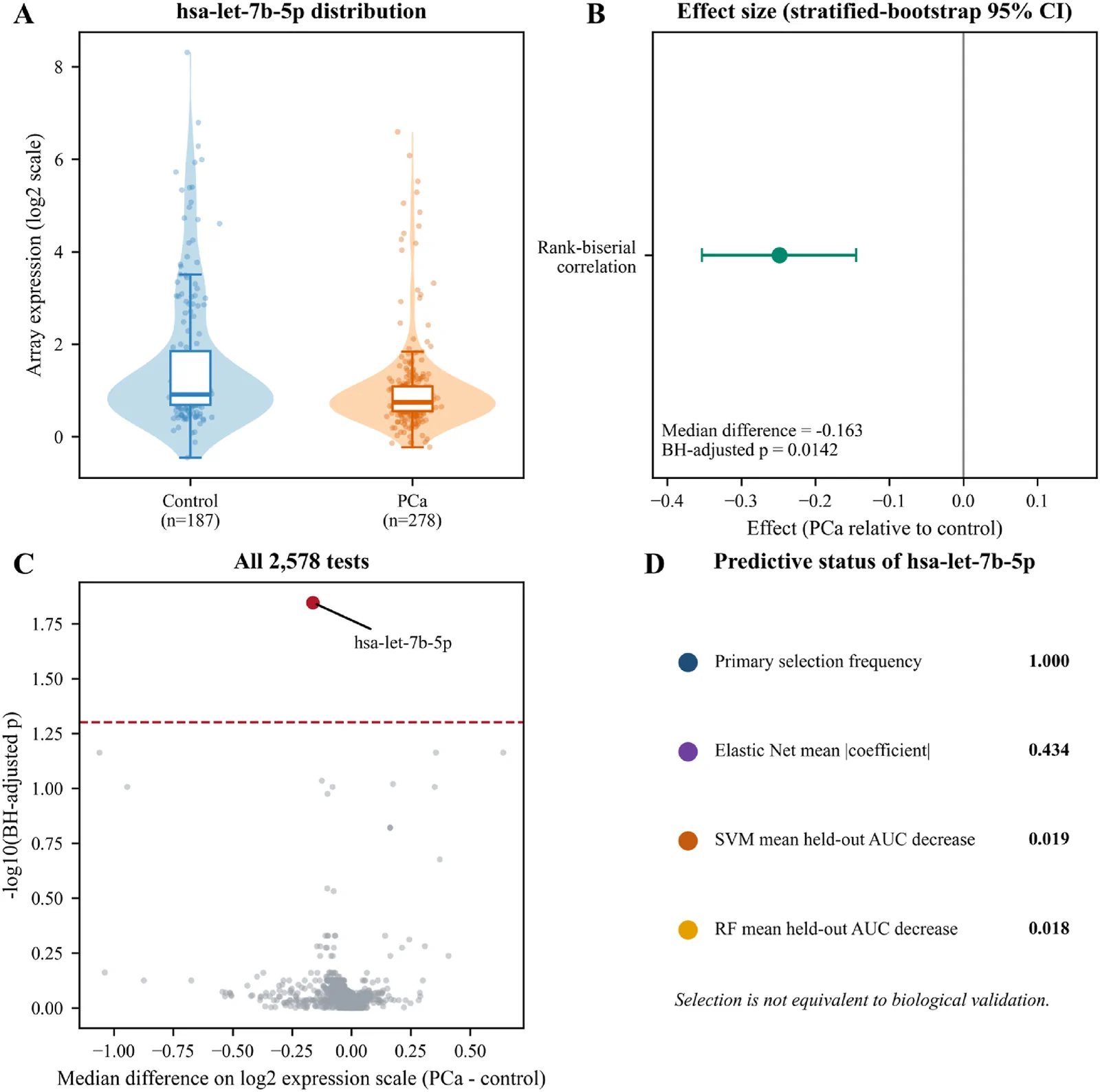
Exploratory hsa-let-7b-5p result and predictive status. (A) Log2-scale expression distributions in 187 control-labelled and 278 positive-labelled records. Biopsy-negative/biopsy-positive labels in the archived plot denote the source-derived classes, not individually reverified pathology results. (B) Rank-biserial effect with stratified-bootstrap 95% interval; the median difference was −0.163 and BH-adjusted p=0.0142. (C) Position of hsa-let-7b-5p among all 2578 Mann-Whitney tests, with the BH threshold. (D) Distinct descriptive indicators of selection recurrence and model-specific predictive contribution. The panels do not establish analytical, functional or clinical validation.

Hsa-let-7b-5p was selected in all five seed-42 outer-training sets and in all 25 outer-training sets across the five partition seeds. Within the fixed-family analyses, its mean Elastic Net coefficient was −0.434, while mean held-out permutation AUC decreases were 0.019 for RBF-SVM and 0.018 for random forest. Differential association, selection recurrence and predictive importance are different properties; their convergence supports prioritisation for replication but not biomarker validation or causal interpretation.

Panel C of Fig. 4 also provides the complete 2578-test landscape: each point pairs the positive-class-minus-control median difference on the log2 expression scale with the BH-adjusted significance level. The horizontal coordinate is a median difference, not a ratio-based fold change. Only hsa-let-7b-5p crossed the adjusted threshold, and this descriptive test landscape did not drive predictive feature selection.

### 3.6 Secondary fixed-family benchmark

In the fixed-family benchmark, Elastic Net yielded AUC 0.714 (95% CI 0.669-0.758) and Brier score 0.218; RBF-SVM yielded AUC 0.737 (0.690-0.781) and Brier score 0.203; random forest yielded AUC 0.746 (0.702-0.789) and Brier score 0.197 (Table 4; Fig. 5). Random forest had the highest observed AUC and lowest Brier score, but paired AUC-difference intervals for Elastic Net versus RBF-SVM, Elastic Net versus random forest, and RBF-SVM versus random forest all included zero. This descriptive result neither establishes AUC superiority within the benchmark nor provides a controlled comparison against the primary fixed-configuration family-selection procedure.

**Table 4:** Secondary and technical sensitivity analyses.

Analysis	Feature universe	Performance (95% CI)	Permitted interpretation
Elastic Net	2578 mature-miRNA probes	AUC 0.714 (0.669-0.758); Brier 0.218 (0.199-0.239)	Secondary fixed-family benchmark.
RBF-SVM	2578 mature-miRNA probes	AUC 0.737 (0.690-0.781); Brier 0.203 (0.187-0.219)	Secondary fixed-family benchmark.
Random forest	2578 mature-miRNA probes	AUC 0.746 (0.702-0.789); Brier 0.197 (0.185-0.210)	Descriptive within-benchmark result; no demonstrated AUC superiority. Not a controlled comparison with the primary fixed-configuration procedure.
Legacy broad-array procedure	All 6658 array probes	AUC 0.756 (0.712-0.799); Brier 0.195 (0.178-0.212)	Technical sensitivity to feature-universe definition; not the primary biological analysis.

All analyses used the same frozen outer assignments. Paired AUC differences (first minus second) were Elastic Net-RBF-SVM, −0.023 (95% CI −0.059 to 0.014); Elastic Net-random forest, −0.032 (−0.071 to 0.006); and RBF-SVM-random forest, −0.009 (−0.045 to 0.026). Every interval included zero. The benchmark used inner grid search; the primary four-family procedure used fixed configurations.

**Fig. 5:**
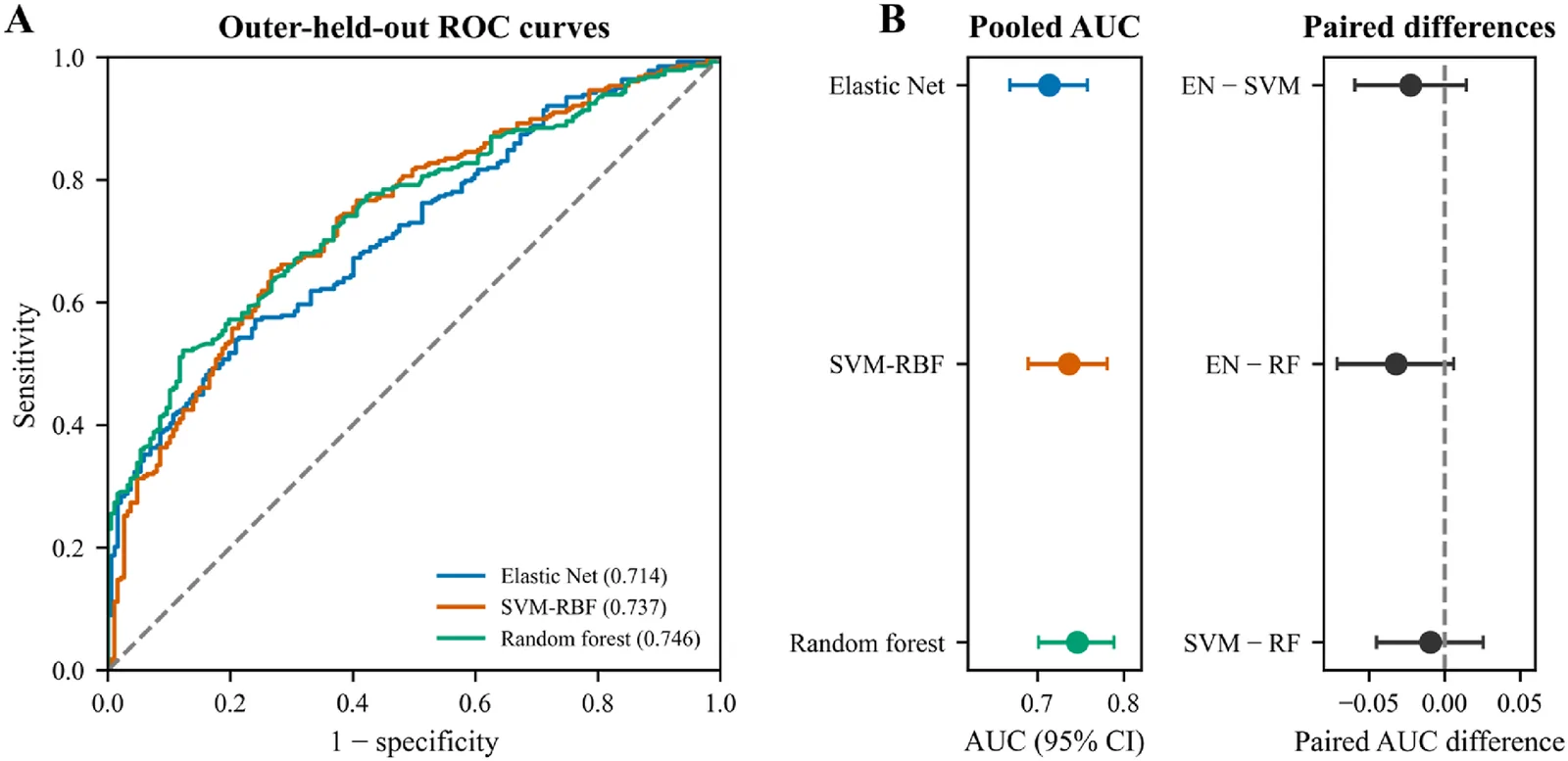
Secondary benchmark discrimination and paired AUC comparisons. (A) Outer-held-out ROC curves for Elastic Net, RBF-SVM and random forest using aligned records and outer partitions. (B) AUC estimates with 95% intervals and paired differences. Random forest had the highest observed AUC (0.746), but every paired AUC-difference interval included zero; the benchmark did not demonstrate statistical superiority in AUC. Comparisons shown here are within this inner-tuned benchmark, not against the primary fixed-configuration procedure.

Internal calibration and threshold metrics differed across the fixed families (Fig. 6). None was externally calibrated. The displays help distinguish a family's observed discrimination from its probability scale and operating characteristics, preventing selection of a “winner” from AUC alone.

**Fig. 6:**
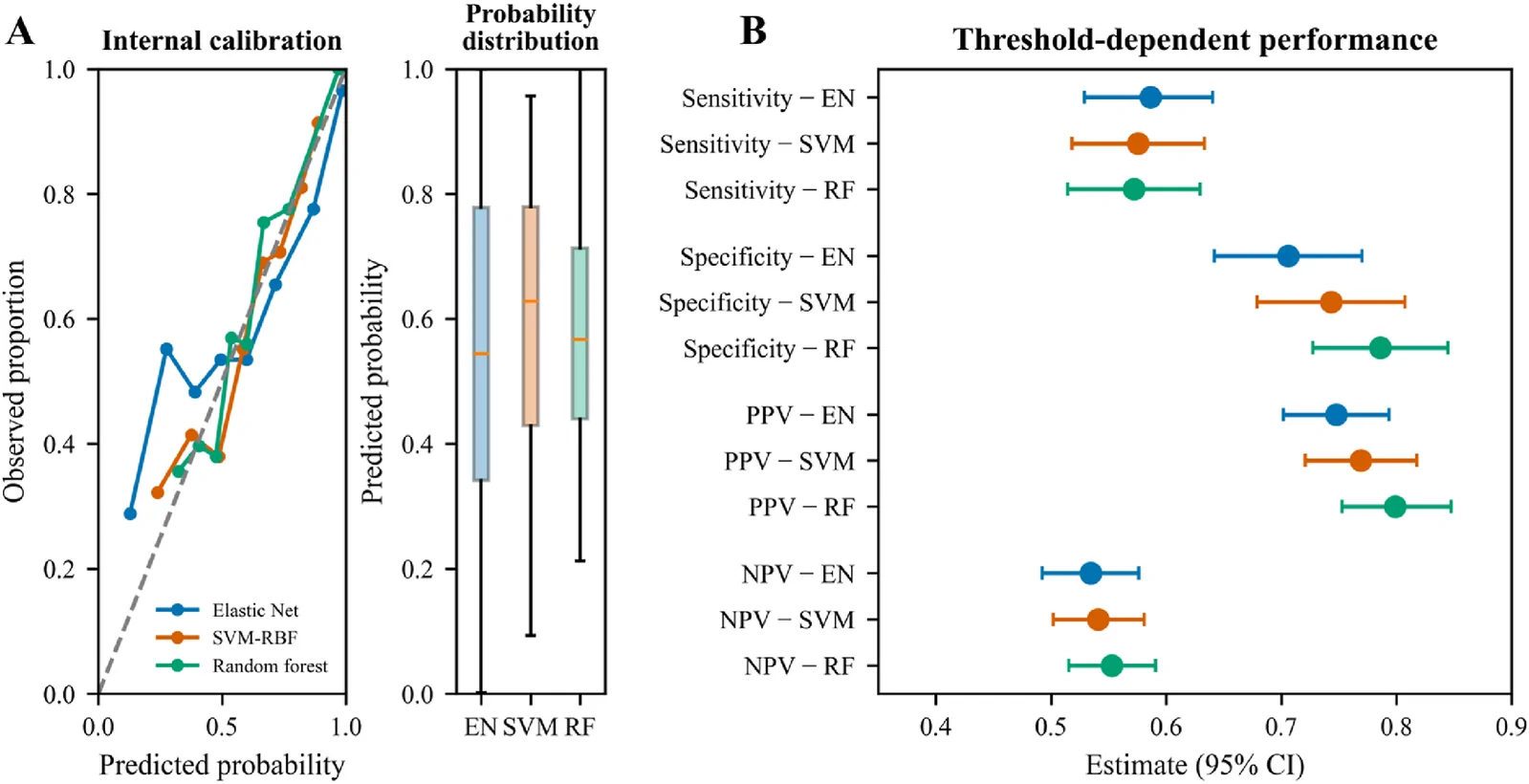
Secondary benchmark calibration and threshold performance. (A) Internal reliability curves and held-out probability distributions for Elastic Net, RBF-SVM and random forest. (B) Sensitivity, specificity, PPV and NPV with 95% intervals after family-specific inner-derived thresholds. All summaries are secondary, internally held out and conditional on the observed case fraction.

The benchmark's partition-level AUCs show that no family dominated every held-out partition (Fig. 7). Here P1-P5 denote the five mutually exclusive outer-held-out partitions: each contains 93 records predicted by a model developed on the complementary 372 records. Configuration identifiers below the plot denote the inner-selected setting for each family and training set; they are technical labels, not biological entities. The mean pairwise Jaccard index of the selected 50-probe sets was 0.539 for each fixed family, a descriptive measure of cross-partition overlap.

**Fig. 7:**
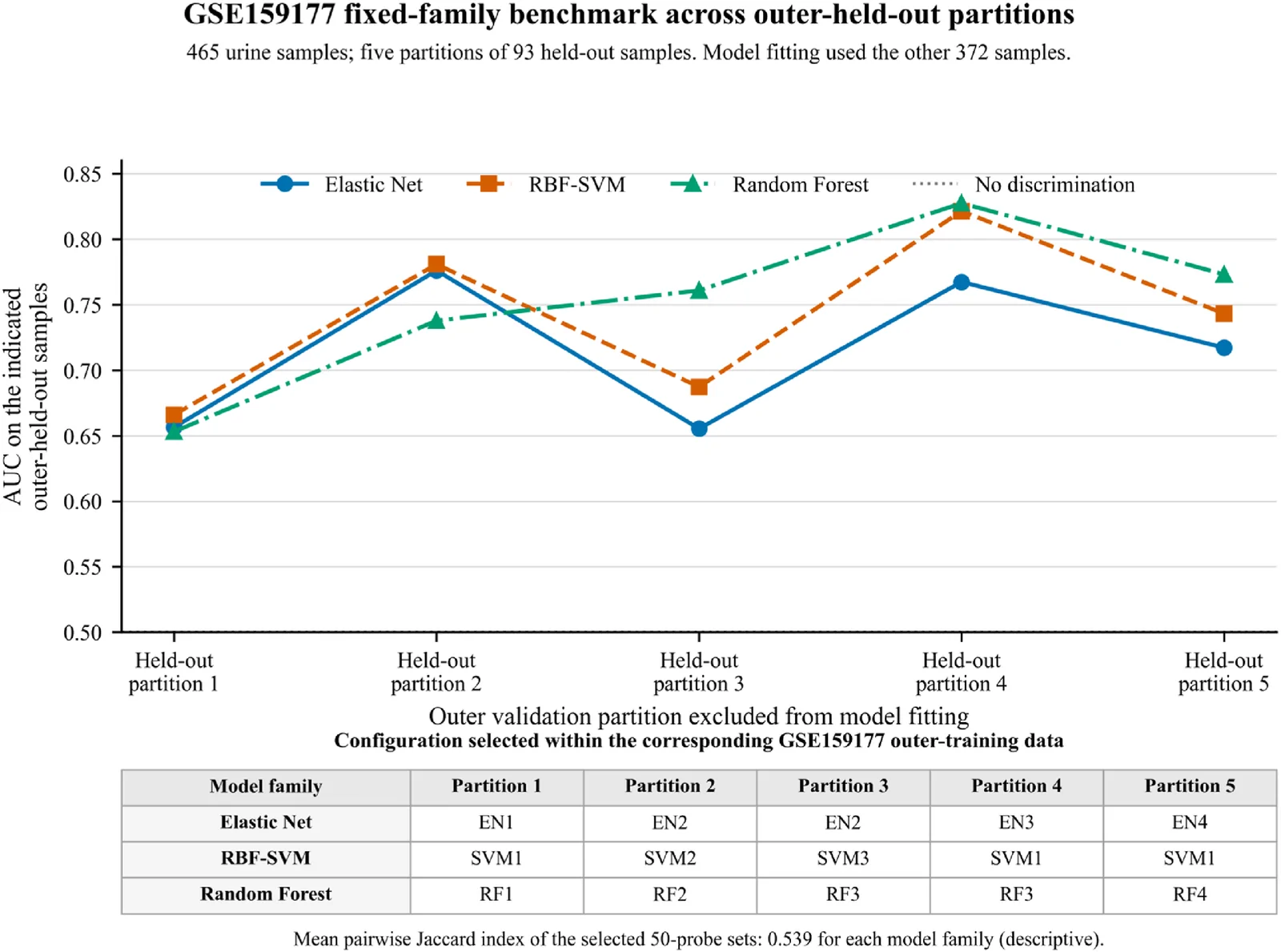
Fixed-family performance across outer-held-out partitions. The lines show AUC for Elastic Net, RBF-SVM and random forest in outer-held-out partitions P1-P5. Each partition contains 93 records; its model was developed on the complementary 372-record outer-training set. The configuration table reports the inner-selected setting for each training set without symbolic separators that imply ordering. Mean pairwise Jaccard overlap of the selected 50-probe sets was 0.539 for each family. These partition-level results are descriptive.

### 3.7 Nested family-selection behaviour

The primary procedure's inner-CV scores varied by outer-training set (Fig. 8). The selected families were random forest twice, RBF-SVM twice and logistic regression once. The margin between the selected and second-ranked family was small in several training sets. These margins quantify conditional selection behaviour, not statistical significance. The horizontal labels “Training set 1-5” mean the complementary development set created when the corresponding outer partition is held out; they are not independent cohorts or chronological stages.

**Fig. 8:**
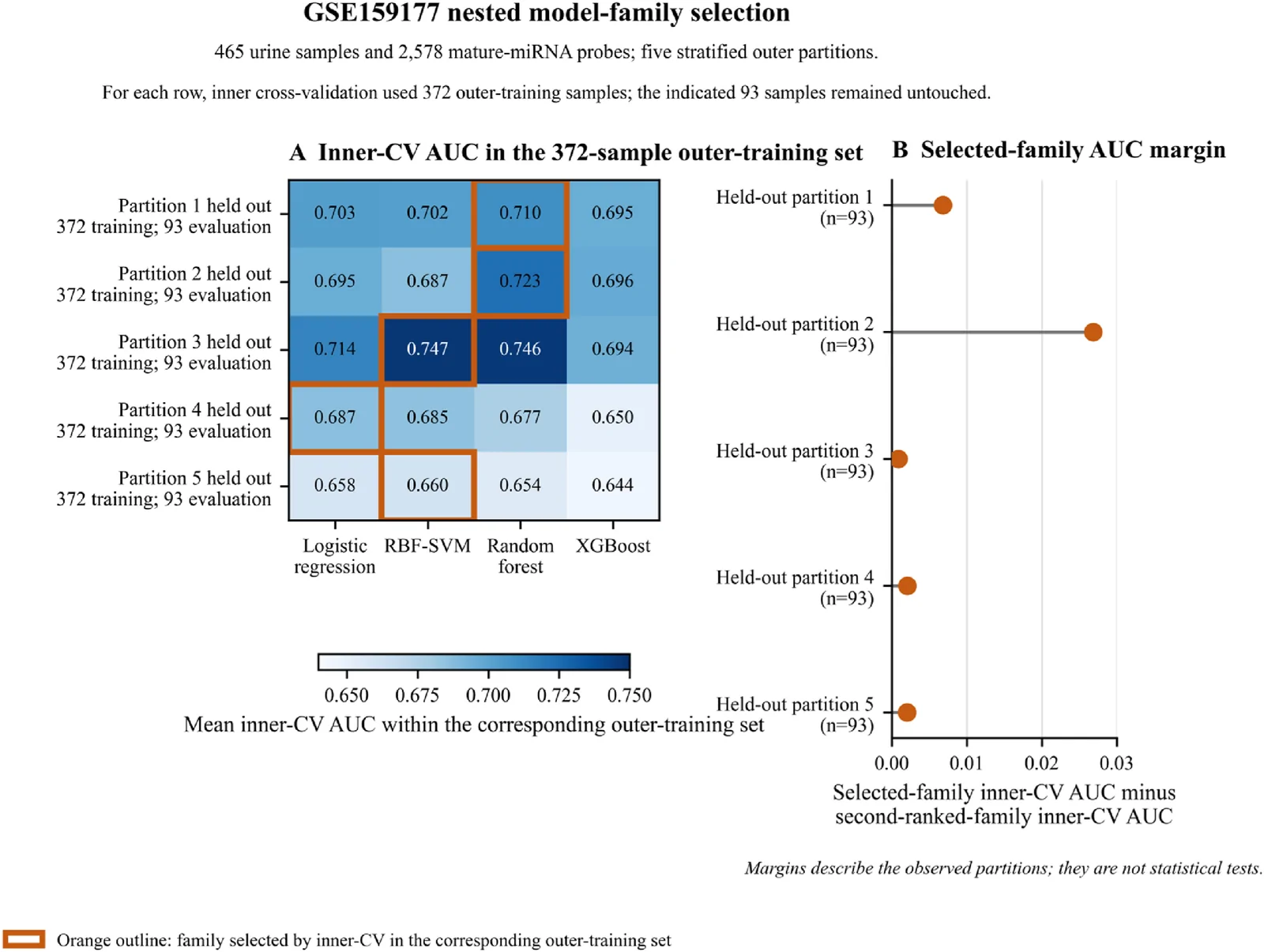
Nested model-family selection within GSE159177 training sets. (A) Inner cross-validated AUC for four candidate families in each outer-training set. Row labels specify which outer-held-out partition was excluded from development. Orange outlines identify the selected family: random forest for training sets 1 and 2, RBF-SVM for sets 3 and 5, and logistic regression for set 4. (B) Difference between the selected and second-ranked inner AUC. Margins are descriptive and are not hypothesis tests.

### 3.8 Predictive importance and stability

Across the five seed-42 outer-training sets, 42 probes were selected at least three times, 34 at least four times and 27 in all five. Fig. 9 displays the highest-ranked probes by held-out permutation contribution, their partition-specific importance and selection frequency. Importance varied across partitions, including near-zero and negative values. Such values describe prediction behaviour in finite held-out subsets; they do not imply absence or reversal of biological function.

**Fig. 9:**
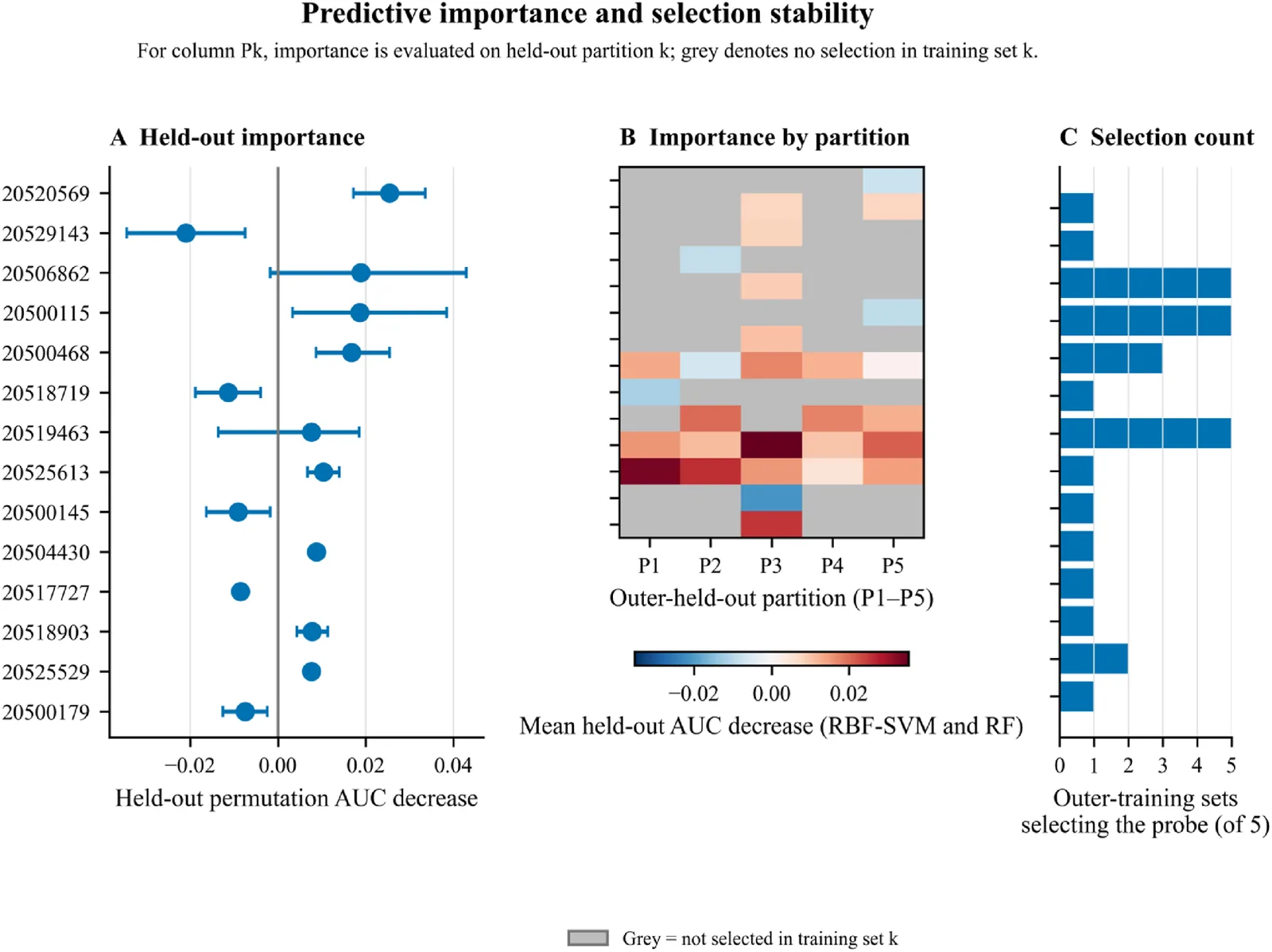
Held-out probe importance and selection stability. (A) Mean and descriptive range of held-out AUC decrease after permutation for recurrent Affymetrix probes. (B) Importance within outer-held-out partitions P1-P5. Grey cells explicitly indicate that the probe was not selected in the corresponding outer-training set; this is distinct from a selected probe with importance near zero. (C) Number of outer-training sets in which each probe was selected. Importance and stability are predictive, descriptive properties and not evidence of biological causality.

### 3.9 Partition sensitivity and exploratory DCA

Across seeds 42, 101, 202, 303 and 404, pooled AUCs were 0.726, 0.681, 0.704, 0.710 and 0.716, respectively. The mean was 0.707 and sample standard deviation 0.017; the range was 0.681-0.726. Selected-family counts also changed by seed, including occasional XGBoost selection. These results show that the canonical seed-42 estimate is conditional on the original recorded partition and should not be interpreted as a partition-invariant property.

The extended DCA (Fig. 10) showed the model and treat-all strategies close together at low thresholds and increasing separation at higher thresholds. The net-benefit scale used the observed 59.8% case fraction. Because no prevalence correction, clinical pathway comparison or external validation was available, the display remains exploratory and does not demonstrate patient benefit.

**Fig. 10:**
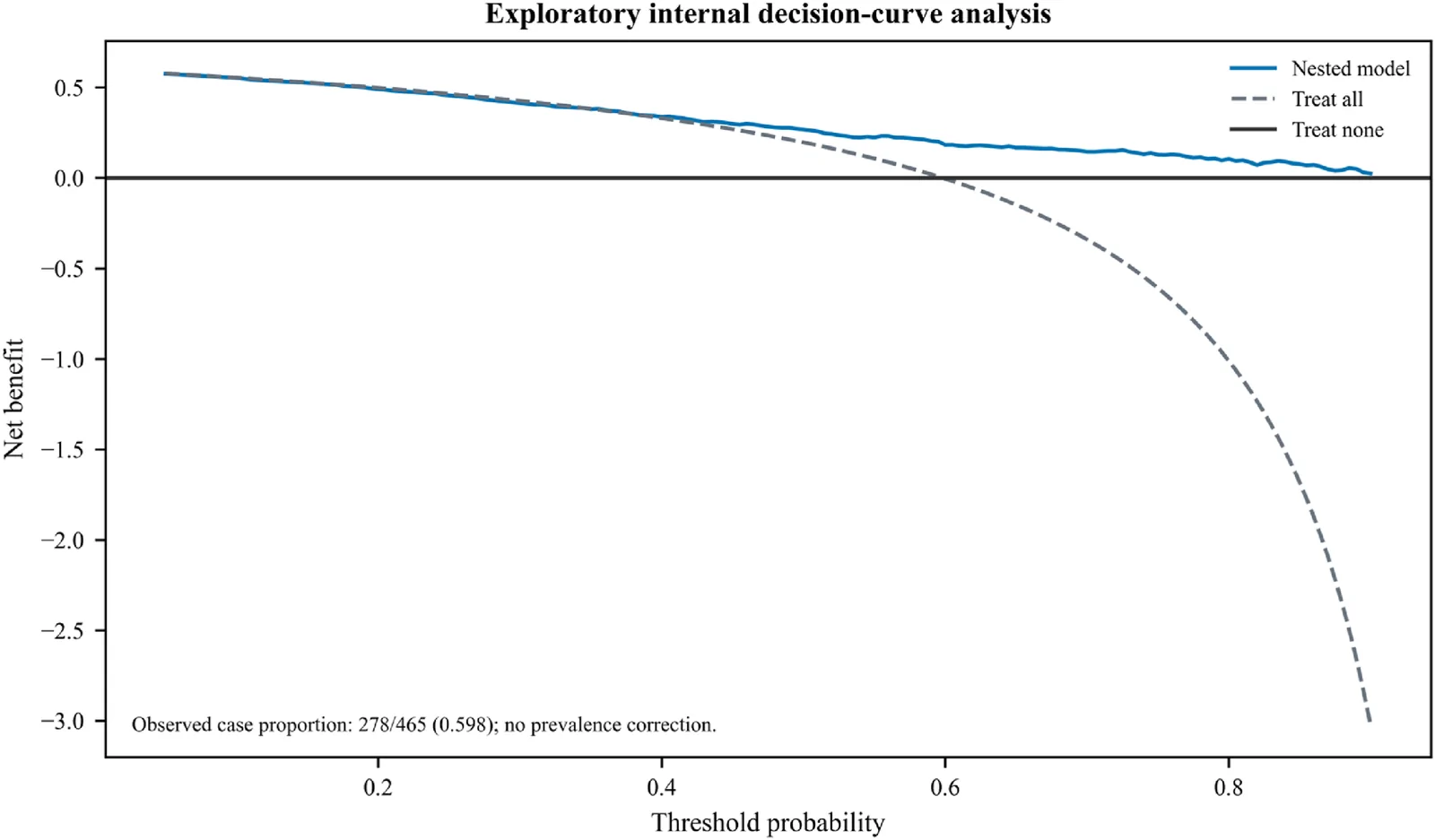
Extended exploratory internal decision-curve analysis. Net benefit is shown for the nested primary procedure, treat-all and treat-none strategies over threshold probabilities 0.05-0.90. This extends the 0.05-0.50 range in Fig. 3. Calculations used the observed 278/465 case fraction without prevalence correction. The analysis is internal and exploratory and does not establish clinical utility.

Restricted to the 448 MU01 records (261 positive-labelled and 187 control-labelled records), pooled held-out AUC was 0.737 (95% CI 0.691-0.783) and Brier score was 0.206 (0.189-0.224). Fourteen of the 17 positive-labelled records with other prefixes were false negatives under the frozen thresholds. These results describe the restricted records only. They do not demonstrate independence from provenance or resolve site-outcome confounding, because no control-labelled records were available in the other prefix groups and the prefixes themselves are not verified site identifiers.

### 3.10 Legacy sensitivity and conditional transportability

The legacy 6658-probe analysis yielded AUC 0.756 (95% CI 0.712-0.799) and Brier score 0.195 (0.178-0.212). Its numerically higher AUC does not replace the primary 0.726 because the input includes biologically heterogeneous and technical probes. This result is a technical sensitivity to the predictor universe.

In the conditional serum transfer experiment, naive logistic regression produced AUC 0.541 (95% CI 0.503-0.581) and CORAL-aligned logistic regression 0.552 (0.515-0.589). Naive random forest produced AUC 0.543 (0.503-0.581) and CORAL-aligned random forest 0.578 (0.541-0.615). The paired CORAL-minus-naive differences were 0.011 (95% CI −0.009 to 0.030) for logistic regression and 0.035 (−0.0003 to 0.070) for random forest (Fig. 11). Both intervals included zero. The result indicates weak cross-source performance and does not establish a benefit of alignment, urinary external validity or clinical generalisability.

**Fig. 11:**
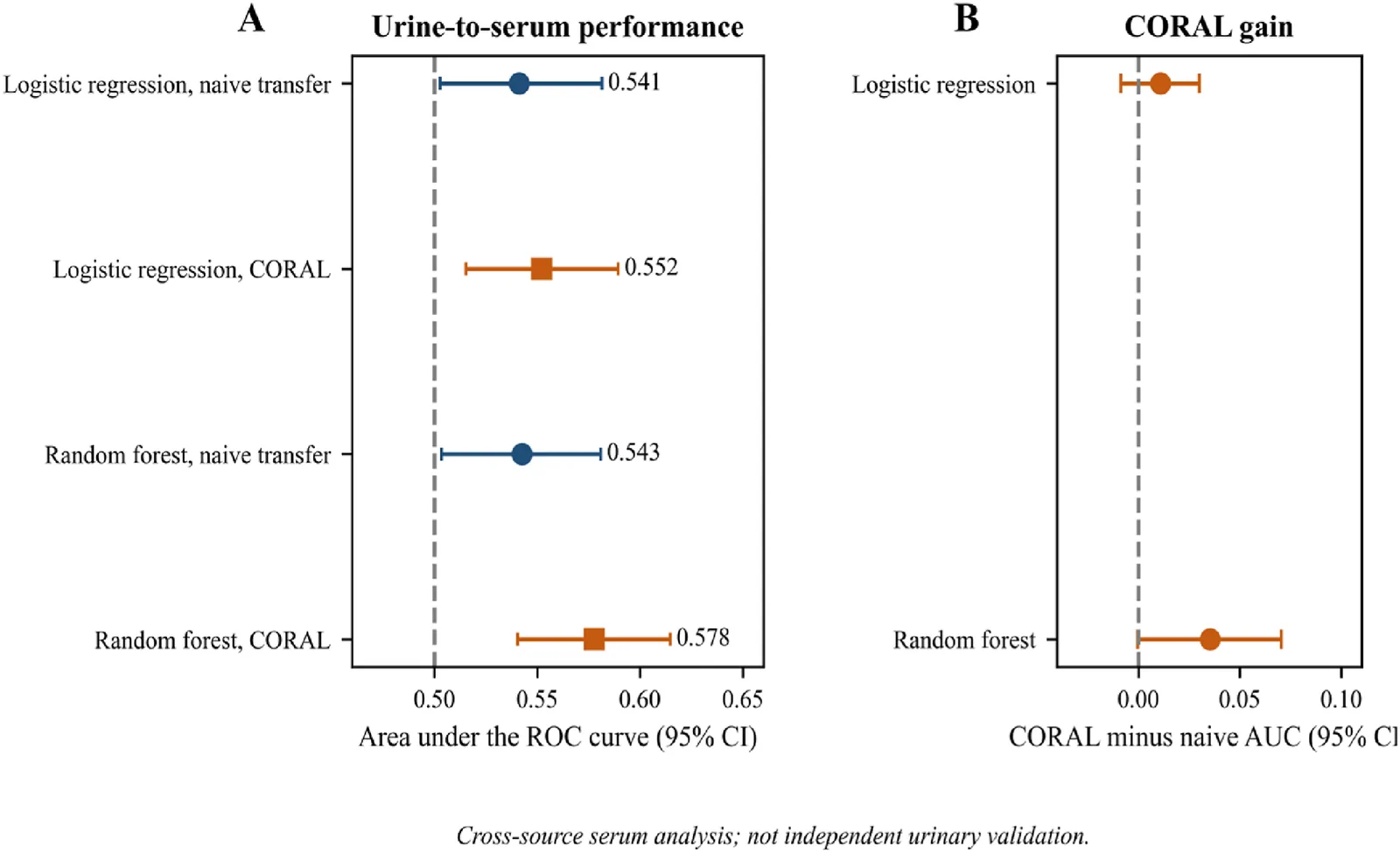
Conditional urine-to-serum transportability stress test. (A) Target-serum AUCs with 95% intervals for source-trained logistic regression and random forest under naive transfer and CORAL alignment. (B) Paired CORAL-minus-naive AUC differences with 95% intervals. The target comprised 1091 GSE112264 serum records. Both difference intervals included zero. This cross-fluid, cross-platform experiment is conditional and is not an independent urinary validation.

### 3.11 Synthesis of the reported evidence

The figures and tables describe complementary analyses of the same public cohort, not independent confirmations. Mean AUC across five recorded repetitions was 0.707 (SD 0.017); the original seed-42 pooled AUC was 0.726. In that repetition, calibration slope was 0.694 and sensitivity was 55.8% at 74.3% specificity, with 123 false negatives. The matched global-selection experiment estimated +0.041 AUC optimism for that particular design. Feature recurrence, secondary family comparisons and the serum stress test remain distinct, internally conditional analyses. hsa-let-7b-5p was the sole BH-significant Mann-Whitney result and a recurrently selected probe, not an independently validated biomarker.

The contribution combines outcome-independent probe annotation, nested predictive evaluation, an empirical leakage comparison and stability assessment in a traceable computational workflow. These complementary analyses provide a basis for prioritising candidates for participant-level urinary replication and targeted experimental evaluation. Their clinical interpretation remains conditional on participant independence and technical provenance; they do not quantify all sources of bias or establish clinical utility.

## 4 Discussion

### 4.1 Principal finding and methodological contribution

This reanalysis found moderate, partition-dependent discrimination under leakage-controlled nested internal validation. Across the five recorded seeds, mean pooled AUC was 0.707 (sample SD 0.017; range 0.681-0.726). The original seed-42 pooled AUC of 0.726 was the highest of these values; its mean fold AUC of 0.730 is a different summary within that repetition. The repetitions reuse the same 465 records and do not establish independent replication. Sensitivity of 55.8% meant that 123 of 278 positive records were missed at the training-derived thresholds. Together with the calibration slope of 0.694 and unresolved data-provenance limitations, these findings do not support clinical triage or absolute-risk estimation.

The central contribution is methodological rather than a claim of a ready biomarker. Annotation defined an explicit mature-miRNA universe; the primary procedure was evaluated as a complete sequence of selection, scaling, family choice, fitting and thresholding; and each record received one held-out prediction. The empirical leakage experiment makes the consequence tangible: global outcome-informed feature selection raised AUC by about 0.041, with a paired interval excluding zero. That invalid result resembles an improvement but is actually evidence of optimistic bias. This finding reinforces prior warnings that microarray classification must nest feature selection within training data [[22], [23], [24]].

### 4.2 Comparison with related urinary studies

The result must be compared with prior urine studies by matching endpoint, compartment and validation design. The GSE159177 source publication developed urinary-exosome small-noncoding-RNA tests in other sample sets and reported detection and grade-related performance [9]. This reanalysis neither reconstructs those proprietary scores nor converts its public labels into a clinically significant-cancer endpoint. It evaluates the deposited Gleason-labelled versus Control-labelled records using a leakage-controlled procedure; individual negative-biopsy status could not be confirmed for the controls.

McKiernan et al. evaluated a three-gene urinary-exosome expression score combined with standard clinical variables for high-grade cancer at initial biopsy and reported an AUC near 0.73 in an independent cohort [4]. The numeric similarity to our AUC is not equivalence: analytes, covariates, outcome, intended use and external-validation status differ. Xu et al. reported improved discrimination when urinary-EV miR-145 was combined with PSA, but used a targeted assay and benign-prostatic-hyperplasia comparators [12]. Ku et al. used acoustic trapping and small-RNA sequencing in a biopsy-referred cohort [13]. Shin et al. addressed metastatic prognosis rather than cancer detection [15]. Each supports urinary RNA as a research direction while illustrating why literature AUCs cannot be pooled informally.

Cell-free urinary and paired urine/plasma EV studies add biological context but not external validation of this classifier [16,42]. The same applies to plasma ctDNA in Fig. 1: evidence that plasma BRCA/ATM alterations can guide testing in advanced prostate cancer does not validate urinary miRNA detection [20,21].

These studies concern different cohorts, assays and intended uses, so they do not establish a general ranking or a common performance estimate for urinary analytes. Our earlier systematic and bibliometric review considered aptamer-based multi-biomarker urinary panels [17]. Within that broader development context, the present study contributes a documented internal evaluation of the source-labelled records, quantifies optimism under a matched leakage experiment, and examines sensitivity to analytic choices. This computational evidence can inform the prioritisation of urinary miRNA candidates for independent assay development; clinical incremental value and economic benefit require separate evaluation.

### 4.3 Biological interpretation of hsa-let-7b-5p

Hsa-let-7b-5p occupies an unusual but still exploratory position. It was the only BH-significant probe and appeared in every primary outer-training feature set, including all 25 sets across five partition seeds. Its negative median difference, negative Elastic Net coefficient and positive held-out permutation contributions provide internally coherent but non-equivalent observations. The Mann-Whitney result concerns group abundance; selection frequency concerns repeated algorithmic eligibility; and permutation importance concerns loss of predictive ranking when a feature is disrupted. All of these observations derive from the same dataset, including the 25 repeated training sets; none constitutes independent validation or proves a molecular mechanism.

The rank shift under Welch and moderated-t sensitivity analyses also discourages overstatement. The repeated within-cohort findings support hsa-let-7b-5p as an exploratory candidate for independent urinary replication using targeted RT-qPCR or ddPCR, rather than as a validated biomarker. Such evaluation should confirm probe identity and sequence, characterise analytical performance and assess incremental value prospectively in an independent urinary cohort.

### 4.4 Algorithm choice, stability and partition dependence

Random forest had the most favourable observed AUC and Brier score in the fixed-family benchmark, but the paired AUC-difference intervals included zero. This does not establish a preferred algorithm. In particular, the benchmark used inner grid search whereas the primary procedure used fixed configurations; their AUCs are not a controlled comparison of model families. The primary selector's use of three different families across five training sets, with small selection margins in some sets, reinforces this caution. Model-family instability is unsurprising when p≫n and candidate procedures have similar inner performance.

Feature recurrence was stronger than complete model-family stability: 27 probes appeared in all five seed-42 selections. Yet even recurrent features showed variable held-out importance. Stability can prioritise measurement development, but it does not by itself demonstrate biological relevance or generalisability. Similarly, the five-seed AUC range of 0.681-0.726 indicates meaningful partition sensitivity. Repeated seeds are not independent cohorts, so the range describes internal variability rather than multiplying the effective sample size.

### 4.5 Calibration, decision consequences and clinical scope

Discrimination, calibration and clinical utility answer different questions. The calibration slope of 0.694 suggests that the spread of predicted risks may be too wide in this dataset. Because no independent cohort was available for recalibration assessment, the model should not be used to provide absolute risk. PPV and NPV are also tied to the observed 59.8% case fraction; values in a screening or referral population would change with prevalence and selection.

The decision curves are correspondingly exploratory. At low thresholds, treat-all can have high net benefit in a case-enriched dataset, and small apparent differences can be sensitive to prevalence and clinical harms. A clinically meaningful DCA would require an intended-use population, relevant comparator pathway, prespecified threshold range and externally validated probabilities. The present curves are useful for checking internal decision behaviour, not for recommending biopsy avoidance.

### 4.6 Transportability and external validation

The serum stress test deliberately challenged the procedure across both specimen and platform. AUCs close to 0.5-0.58 and difference intervals crossing zero show that source-trained signals did not transport convincingly. CORAL uses unlabeled target covariance structure, but its apparent gains were uncertain. This analysis should not be called external urinary validation: GSE112264 contains serum, has a different sample composition and measures a related but biologically distinct compartment [40].

True external validation requires a locked model and preprocessing rule applied without redevelopment to an independent participant-level urinary cohort representing the intended clinical setting. It should preserve the same outcome definition or explicitly address a new one. Recalibration, if needed, must be separated from evaluation, and performance should be accompanied by confidence intervals and clinically relevant subgroup analyses.

### 4.7 Strengths and limitations

Strengths include complete reconciliation of 465 unique held-out predictions; an explicit, outcome-independent mature-miRNA annotation; a frozen five-partition information barrier; separation of primary, descriptive, secondary and conditional analyses; paired comparisons on aligned observations; empirical quantification of leakage; record-level uncertainty; partition-sensitivity analysis; held-out permutation importance; and a reproducibility package containing scripts, inputs, seeds, predictions, logs and hashes. The analysis also avoids relabelling serum or literature evidence as urinary validation.

Several limitations remain. First, all primary evidence comes from one public dataset; the seed-42 result was the highest of five recorded repetitions. Second, bootstrap intervals conditional on frozen predictions omit full split and training variability. Third, no participant-grouping key was available, so repeated participants across partitions and resulting optimism cannot be excluded. Fourth, public Control labels do not independently verify negative biopsies for individual records. All control-labelled records share the MU01 prefix; the descriptive MU01 restriction does not resolve provenance-outcome confounding, and prefix-to-site definitions are unconfirmed. Fifth, recovered scan dates show outcome imbalance across acquisition periods, but extraction and hybridisation batches remain unknown; processing-directory groups and matrix completeness do not establish technical reliability. Sixth, clinical covariates were insufficient to evaluate incremental value beyond age, PSA or MRI, clinically significant disease or subgroup fairness. Seventh, EV isolation and purity were not independently re-characterised. Finally, no independent urinary validation, targeted PCR assay or functional experiment was performed; secondary and cross-source analyses do not substitute for these requirements.

### 4.8 Required next steps

Future development should define the intended clinical use before model locking. A participant-level urinary cohort should include biopsy outcome and clinically significant disease variables, standardise collection and EV processing, and avoid multiple samples crossing validation partitions. A small, assay-compatible feature panel should be prespecified from the stable candidates and confirmed by targeted RT-qPCR or ddPCR. The complete locked pipeline-normalisation, feature identities, coefficients or trees, and threshold-should then undergo external validation with discrimination, calibration-in-the-large, calibration slope, threshold errors and DCA at a credible target prevalence. hsa-let-7b-5p should be treated as a separate replication hypothesis until analytical and experimental validation are complete.

In that independent urinary validation, the locked model will first be evaluated without recalibration. If recalibration is required, the planned approaches are intercept-only recalibration for a shift in average predicted risk and logistic recalibration of both intercept and slope for miscalibration of risk level and spread [33]. The latter uses logit (p_updated_) = a + b × logit (p_original_). Recalibration parameters will be estimated on data reserved for updating, and performance of the updated model will be assessed on separate data; these participants will not be reused to claim independent post-update performance. The internal slope of 0.694 will not simply be imposed on a new population. No recalibration or improvement in external performance is claimed in the present study.

## 5 Conclusions

Under leakage-controlled nested validation, urinary extracellular-vesicle-associated mature-miRNA profiles provided moderate discrimination between positive-labelled records and control-labelled comparators. Mean pooled AUC across the five recorded repetitions was 0.707 (sample SD 0.017; range 0.681-0.726); the originally reported seed-42 AUC was 0.726. Sensitivity was 55.8% in that analysis, with 123 false negatives among 278 positive records. Calibration was imperfect and participant independence remained unresolved. The leakage experiment showed that global feature selection could inflate apparent discrimination. Together, the annotation, nested evaluation, leakage quantification and stability analyses provide a reproducible basis for prioritising urinary miRNA candidates. hsa-let-7b-5p is an exploratory candidate for independent urinary replication using a locked targeted assay; experimental confirmation and participant-level validation remain necessary before clinical translation.

## Ethics approval and consent to participate

Not applicable. This study was a secondary analysis of publicly available, de-identified data. No new participant recruitment, specimen collection or intervention was performed.

## Consent for publication

Not applicable.

## Availability of data and materials

GSE159177 is publicly available from NCBI GEO at https://www.ncbi.nlm.nih.gov/geo/query/acc.cgi?acc=GSE159177 [8]. GSE112264, used only for the conditional serum stress test, is available at https://www.ncbi.nlm.nih.gov/geo/query/acc.cgi?acc=GSE112264 [41]. Frozen derived predictions, fold assignments, annotation tables, metrics and figure source data accompany this editorial package. No participant-level identifier was available.

## Code availability

The reproducibility package contains analysis and figure scripts, imported helpers, locked environments, seeds, execution logs and SHA-256 manifests. No public repository or archival DOI is claimed at this stage. The package is intended for independent computational audit before deposition.

## Authors' contributions (CRediT)

A.S.:Conceptualization, Formal analysis, Writing - original draft. N.R., I.D., I.B. and H.M.: Methodology and interpretation. A.A.: Supervision. All authors: Writing - review and editing. All authors reviewed and approved the manuscript.

## Declaration of generative AI and AI-assisted technologies in the writing process

During the preparation of this work, the authors used generative AI chatbots for language editing and polishing. The authors have thoroughly reviewed and edited the output and take full responsibility for the content of this publication.

## Funding

This research received no external funding.

## Declaration of competing interest

The authors declare that they have no competing interests.

## Data Availability

Research Link Provided

## List of abbreviations

AUC, area under the receiver-operating-characteristic curve; BH, Benjamini-Hochberg; CI, confidence interval; CORAL, correlation alignment; CV, cross-validation; DCA, decision-curve analysis; ddPCR, digital droplet polymerase chain reaction; DOR, diagnostic odds ratio; EV, extracellular vesicle; GEO, Gene Expression Omnibus; LR, logistic regression; miRNA, microRNA; MISEV, Minimal Information for Studies of Extracellular Vesicles; NPV, negative predictive value; PCa, prostate cancer; PPV, positive predictive value; PSA, prostate-specific antigen; RBF, radial basis function; RF, random forest; RNA, ribonucleic acid; ROC, receiver-operating-characteristic; RT-qPCR, reverse-transcription quantitative polymerase chain reaction; SVM, support-vector machine; XGB, XGBoost.
